# Applications of Genome Sequencing in Infectious Diseases: From Pathogen Identification to Precision Medicine

**DOI:** 10.3390/ph18111687

**Published:** 2025-11-07

**Authors:** Gulam Mustafa Hasan, Taj Mohammad, Anas Shamsi, Sukhwinder Singh Sohal, Md. Imtaiyaz Hassan

**Affiliations:** 1 Department of Basic Medical Science, College of Medicine, Prince Sattam Bin Abdulaziz University, Al-Kharj 11942, Saudi Arabia; mgulam@gmail.com; 2 Centre for Interdisciplinary Research in Basic Sciences, Jamia Millia Islamia, New Delhi 110025, India; tajmohammad.tk@gmail.com; 3 Centre of Medical and Bio-Allied Health Sciences Research, Ajman University, Ajman P.O. Box 346, United Arab Emirates; 4 Respiratory Translational Research Group, Department of Laboratory Medicine, School of Health Sciences, College of Health and Medicine, University of Tasmania, The Shed Building, 80 Cimitiere Street, Launceston, TAS 7250, Australia; sukhwinder.sohal@utas.edu.au

**Keywords:** precision medicine, genome sequencing, metagenomics, antimicrobial resistance, pathogen surveillance, drug targets

## Abstract

**Background:** Genome sequencing is transforming infectious-disease diagnostics, surveillance, and precision therapy by enabling rapid, high-resolution pathogen identification, transmission tracking, and genomic-informed antimicrobial stewardship. **Methods:** We review contemporary sequencing platforms (short- and long-read), targeted and metagenomic approaches, and operational workflows that connect laboratory outputs to clinical and public health decision-making. We highlight strengths and limitations of genomic AMR prediction, the role of plasmids and mobile elements in resistance and virulence, and practical steps for clinical translation, including validation, reporting standards, and integration with electronic health records. **Results:** Comparative and population genomics reveal virulence determinants and host–pathogen interactions that correlate with clinical outcomes, improving risk stratification for high-risk infections. Integrating sequencing with epidemiological and clinical metadata enhances surveillance, uncovers cryptic transmission pathways, and supports infection control policies. Despite these advances, clinical implementation faces technical and interpretative barriers, as well as challenges related to turnaround time, data quality, bioinformatic complexity, cost, and ethical considerations. These issues must be addressed to realize routine, point-of-care sequencing. **Conclusions:** Emerging solutions, including portable sequencing devices, standardized pipelines, and machine-learning models, promise faster, more actionable results and tighter integration with electronic health records. The widespread adoption of sequencing in clinical workflows has the potential to shift infectious disease management toward precision medicine, thereby improving diagnostics, treatment selection, and public health responses.

## 1. Introduction

The rapid and precise identification of infectious agents has always been at the forefront of clinical care, infection prevention, and public health response [1]. Common diagnostic methods, such as culture, microscopy, and targeted molecular assays, remain essential; however, each has its own inherent limitations [2,3]. Traditional methods, such as culture, microscopy, and targeted molecular assays, have proven indispensable but are limited by turnaround time, sensitivity, and the requirement for prior pathogen knowledge [4,5]. Technical advances in short- and long-read platforms, together with culture-independent metagenomic sequencing, have revolutionized pathogen identification and resistance tracking across clinical and public health settings [1,6,7]. The clinical and public health impact of sequencing is already significant and continues to increase.

Whole-genome sequencing (WGS) provides unambiguous strain typing and high-resolution phylogenetic data for use in outbreak detection, source attribution, and transmission mapping, both within hospitals and communities, as well as across national borders [8]. The direct identification of resistance genes and predictive markers at the genomic level holds the potential for rapid, evidence-based antimicrobial decision-making and stewardship, leading to improved patient outcomes and reduced pressure for the selection of AMR [9]. By using comparative genomics to define virulence determinants and mechanisms of immune evasion, as well as genetic correlates of clinical severity, vaccine design and therapeutic development can be informed and accelerated. Pathogen sequence data paired with epidemiological metadata and electronic health record data facilitate risk stratification. They can help guide precision approaches to infectious disease management, such as targeted prophylaxis and personalized therapy options [10,11].

However, translating sequencing into routine clinical use faces a range of technical, operational, and ethical challenges. Developing metagenomic technologies that can shorten assay time to clinically relevant decision windows remains a priority. Equally important is the ability to distinguish infection from colonization or background contamination, and to interpret the clinical significance of genomic variants. Detection of novel or poorly characterized determinants requires curated databases and standardized interpretation frameworks. Institutional bioinformatics expertise and computational infrastructure vary, and discrepancies in laboratory accreditation, regulatory oversight, and reimbursement policies limit widespread implementation [12,13]. This review summarizes sequencing technologies, clinical and public health applications, and implementation challenges, with a focus on translational links between laboratory genomics, clinical practice, and public health.

## 2. Sequencing Technologies and Methodological Advances

Sequencing have shifted over the past decade from an esoteric research tool to a set of pragmatic tools applicable to clinical and public health questions. Current sequencing methods for infectious diseases can be broadly organized into platform technologies, targeted versus untargeted assays, and downstream analytical workflows [14].

### 2.1. Sequencing Platforms and Assay Selection

**Figure 1:**
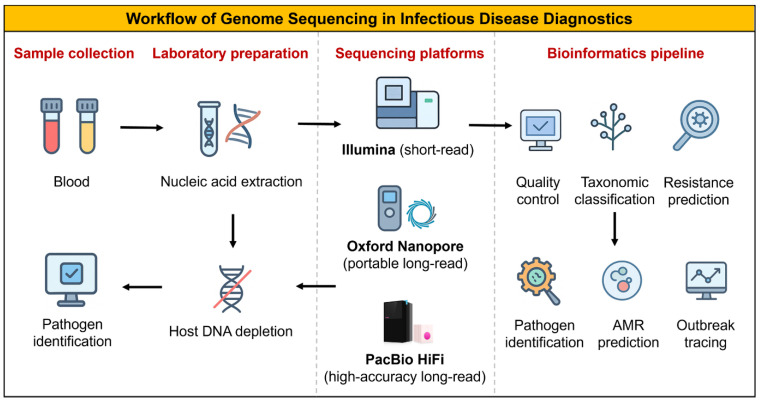
Workflow of genome sequencing in infectious disease diagnostics. The process begins with specimen extraction, followed by pathogen enrichment and library preparation and culminates in sequencing and quality control (QC) assessment. The resulting data are then analyzed through taxonomic classification and antimicrobial resistance (AMR) detection, culminating in clinical interpretation and integration of the final report into the electronic health record (EHR) system.

Short-read platforms (Illumina) offer high per-base accuracy and cost-efficiency for routine bacterial WGS and viral surveillance. In contrast, long-read platforms (PacBio HiFi, ONT) resolve plasmids, structural variation, and full operons, crucial when genomic context determines phenotype [1,15]. Targeted assays, such as amplicon panels or hybrid capture, increase sensitivity and speed for known targets, while untargeted metagenomics (mNGS) supports hypothesis-free detection in culture-negative or atypical infections (Table 1). Hybrid short/long read strategies often provide the best balance of accuracy and structural resolution when plasmid-resolved inference or mobile-element mapping is required [16,17]. The sequential steps from clinical specimen to actionable sequencing report are illustrated in Figure 1.

### 2.2. Enrichment, Library Preparation, and Informatics

**Figure 2:**
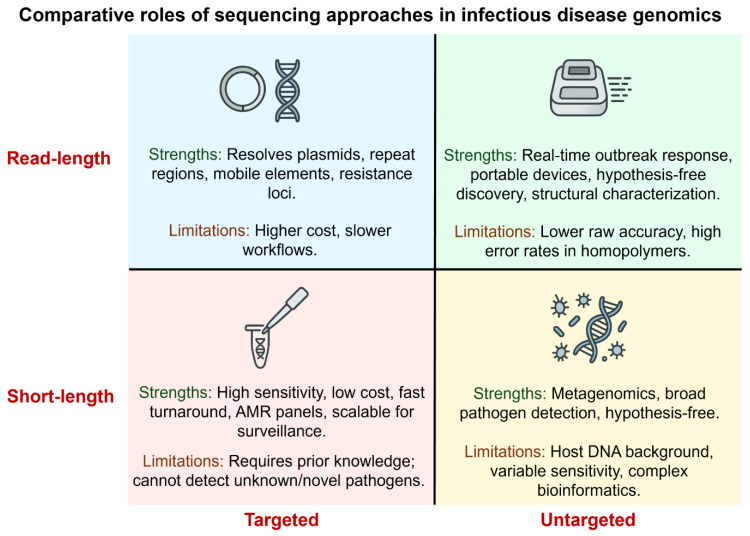
Comparative roles of sequencing approaches. A two-axis framework highlights differences between targeted and untargeted assays on one axis and between short- and long-read technologies on the other. Targeted short-read assays deliver high sensitivity for predefined loci, while untargeted short-read metagenomics enables hypothesis-free detection. Targeted long-read strategies resolve plasmids and complex loci, whereas untargeted long-read sequencing supports real-time outbreak response and structural characterization.

Optimized enrichment strategies and standardized library preparation are essential to enhance pathogen detection in low-biomass clinical samples. These enrichment strategies, ranging from selective lysis and rRNA depletion to probe-based or on-device enrichment [1,15]. Common enrichment strategies, such as selective lysis, rRNA depletion, probe-based capture, or adaptive sampling, enhance pathogen signal and improve detection sensitivity. Bioinformatic pipelines encompass preprocessing, classification or assembly, resistance-gene detection, and variant annotation, culminating in clinically interpretable reports. Version-controlled databases (e.g., CARD, AMRFinder, ResFinder) and transparent documentation of thresholds ensure reproducibility and reliability. Machine-learning tools complement rule-based approaches but require rigorous validation against phenotypic data before clinical use [18]. Targeted short-read assays remain optimal for routine surveillance, while metagenomic sequencing and hybrid strategies are reserved for cases with unresolved diagnosis or structural complexity (Figure 2).

## 3. Pathogen Identification and Surveillance

Genome sequencing can provide the most immediate clinical and public health benefits, including accurate pathogen detection and the contextualization of isolates within an epidemiological setting. Here, we discuss clinical diagnostics, including both targeted and metagenomic approaches; genomic epidemiology for outbreak detection and source attribution; population-level surveillance, such as wastewater genomics; and practical aspects, with a particular emphasis on MDR *Klebsiella*. In each subsection, we discuss the advantages of sequencing compared to the standard of care, the limitations of sequencing, and the conversion of genomic data into actionable elements to guide clinical decision-making [16].

### 3.1. Clinical Pathogen Identification

Targeted sequencing enables high-sensitivity detection of predefined pathogens or resistance loci, while metagenomic sequencing supports hypothesis-free identification across bacteria, viruses, fungi, and parasites [19]. In practice, most laboratories employ hybrid workflows that combine rapid targeted assays for common pathogens with metagenomic sequencing for unresolved or atypical cases, maximizing clinical yield while controlling cost and turnaround time [20]. Pragmatic challenges are poor sensitivity in the setting of abundant host nucleic acid, inability to distinguish colonization/commensal flora from invasive disease, and a higher burden of bioinformatic analysis and clinical correlation [21]. Most laboratories have developed hybrid workflows that incorporate rapid targeted assays for common, high-probability pathogens, reserving mNGS diagnostics for cases that remain unresolved or in which broad pathogen discovery is necessary, as the clinical yield increases [20,22,23] (Table 2).

### 3.2. Genomic Epidemiology

Whole-genome sequencing enables fine-scale resolution of transmission chains across healthcare and community settings, surpassing conventional typing methods [24]. Integration of genomic data with epidemiological metadata, such as timing, patient movement, and exposure, accelerates outbreak tracing and guides targeted infection control interventions. Standardized analysis frameworks are essential to avoid overinterpretation and ensure consistent resolution thresholds [25]. Additionally, researchers should consider pathogen-specific mutation rates, within-host diversity, and plasmid transmission, which can decouple resistance genotypes from chromosomal phylogeny.

### 3.3. Population-Scale Surveillance

In addition to individual and outbreak diagnosis, sequencing enables continuous surveillance of pathogen evolution, resistance emergence, and geographic spread. These genomic surveillance programs, both national and regional, for influenza, SARS-CoV-2, foodborne pathogens, and priority AMR organisms, provide an evidence base for public health policy, e.g., vaccine strain selection, infection control guidance, and stewardship priorities [26]. National and regional genomic surveillance programs for influenza, SARS-CoV-2, and priority AMR organisms inform infection-control policies and vaccine strain selection. Wastewater sequencing detected rising Omicron lineage prevalence earlier than clinical sampling in several regions, prompting targeted clinical testing and public health interventions [27]. Global coordination efforts such as the WHO GLASS Genomic Module, CDC Pathogen Genomics Program, and EMBL-EBI Pathogenwatch framework now standardize genomic data submission, ensuring comparability and rapid dissemination of AMR and outbreak information worldwide. For *Klebsiella pneumoniae*, genomic surveillance has revealed global spread of carbapenemase plasmids and high-risk lineages, informing infection-control strategies [28,29].

## 4. Antimicrobial-Resistance Prediction from Genomes

Genomic prediction of antimicrobial resistance from pathogen genomes is among the highest-value clinical applications of sequencing, as it can shorten the time to effective therapy, guide antimicrobial stewardship, and detect and track high-risk resistance determinants. However, the promise of this approach is limited by several factors [26]. The accuracy of predictions depends on the quality of the reference knowledge that links sequences to phenotypes. Genomic prediction of AMR is one of the most clinically impactful applications of sequencing, offering faster therapy guidance and resistance tracking. However, it is constrained by genotype–phenotype variability and the quality of reference databases.

### 4.1. Reference Resources and the Genotype-Phenotype Framework

Accurate AMR prediction depends on curated resistance databases linking specific genes, alleles, and mutations to phenotypes [30]. Several well-known, actively curated resources are widely used in clinical and research pipelines for antimicrobial resistance analysis. The Comprehensive Antibiotic Resistance Database (CARD) provides an ontology-based collection of resistance determinants and detection models [31]. The Center for Genomic Epidemiology’s ResFinder and PointFinder systems identify acquired genes and chromosomal mutations associated with resistance. NCBI’s AMRFinderPlus uses a curated reference gene catalog and Hidden Markov Models to detect resistance genes and relevant mutations [32,33]. These resources vary in scale, scope of curation, and update frequency, and many clinical pipelines cross-reference two or more databases for increased sensitivity and interpretability.

### 4.2. Algorithms and Analytic Approaches: Rule-Based, Comparative, and ML Methods

**Figure 3:**
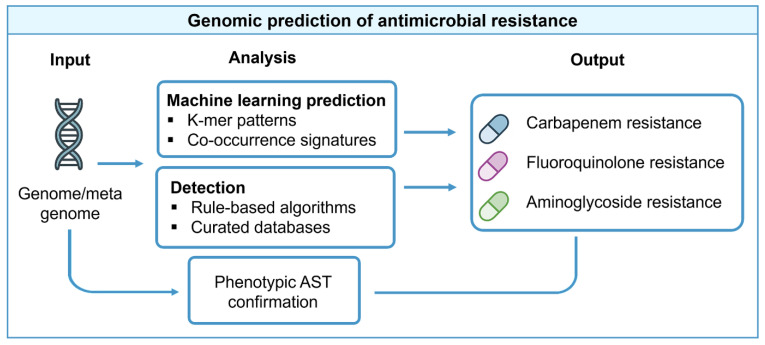
Genomic prediction of AMR. Genome or metagenome sequences enter rule-based pipelines that detect known genes and mutations, supplemented by machine-learning models that capture complex genomic patterns. Outputs predict resistance to antibiotic classes such as carbapenems, fluoroquinolones, or aminoglycosides. Feedback from phenotypic testing informs continuous refinement of the database and improvement of the model.

Rule-based inference remains standard for well-characterized mechanisms such as beta-lactamases and target-site mutations, offering transparency and ease of validation. It remains the backbone of routine surveillance tools. Machine-learning models, including DeepARG and related frameworks, augment rule-based pipelines by capturing non-obvious genomic patterns. However, their clinical utility depends on large annotated training datasets, external validation, and transparent performance metrics [34]. The workflow linking genomic inputs to resistance phenotype prediction is illustrated in Figure 3. Several bioinformatic resources have been developed to support resistance prediction from sequence data, as summarized in Table 3.

### 4.3. Clinical Implementation and Validation

Clinical implementation of genomic AMR prediction requires analytical and clinical validation against phenotypic gold standards, with stringent quality assurance, contamination control, and version tracking of databases and pipelines [35]. Effective reporting frameworks should clearly state the detected genes, predicted phenotypes, and confidence levels, and should be supported by provenance details, such as database versions and thresholds. Stepwise adoption, beginning with surveillance and infection control before clinical therapy guidance, is recommended.

### 4.4. Limitations, Common Pitfalls, and Opportunities for Improvement

Three limitations repeatedly restrict the widespread substitution of phenotypic AST with genomic prediction. First, although determining the presence or absence of a sequence is straightforward, several factors can cause discrepancies between the genotype and phenotype [36]. Second, plasmids and their capacity for horizontal transfer allow resistance genes to move independently of the chromosomal background, further complicating prediction [37]. The third source of ambiguity is metagenomic context: the absence or presence of a resistance gene in a sample may not always be informative about the gene-carrying organisms that cause disease, i.e., the resistance might be present in non-pathogenic or dead cells. Closing these gaps is an active area of research. Genotype–phenotype discordance arises from regulatory variation, gene copy number, plasmid mobility, and novel mechanisms that are not present in reference databases. Therefore, a hybrid model combining rapid genomic prediction for stewardship with phenotypic AST for confirmation offers a pragmatic path toward reliability [38]. Standardized ontologies such as the Antibiotic Resistance Ontology (ARO) and curated repositories like GAARD improve interoperability and facilitate global benchmarking [39].

## 5. Virulence Determinants and Pathogenesis

Characterizing the pathogenic potential associated with genomic variation is crucial for utilizing sequencing to inform clinically relevant risk stratification, targeted therapy, and prevention. Sequencing facilitates systematic discovery and surveillance of virulence factors and their genomic contexts, enabling correlations with disease severity and transmission [40,41].

### 5.1. Genetic Architecture of Virulence

Pathogen virulence is an emergent property of both conserved core functions and a highly variable accessory genome. The mosaic nature of its architecture implies that clinical behavior can differ dramatically between closely related strains, driven by the gain or loss of a small number of high-impact loci. Pathogen virulence reflects both conserved core functions and a variable accessory genome often carried on plasmids or genomic islands; pangenome analyses therefore help identify conserved targets for diagnostics and lineage-associated features that explain the emergence of severe disease. High-quality assemblies that capture plasmids and repeats are essential to map these loci accurately [42,43].

### 5.2. Mobile Elements, Plasmids, and the Coupling of Resistance with Virulence

Mobile genetic elements, including plasmids, facilitate the rapid spread of both resistance and virulence gene determinants. The co-localization of these features on the same mobile platform, particularly plasmids, carries significant clinical implications [44]. Mobile genetic elements, including conjugative plasmids, integrative elements, and phages, drive rapid co-transmission of virulence and resistance loci. The co-location of virulence and AMR genes on mobile elements accelerates the emergence of high-risk convergent strains; therefore, plasmid and mobile-element reconstruction (often via long-read or hybrid assemblies) is a priority for genomic surveillance [45,46].

### 5.3. From Genotype to Mechanism: Virulence Factors, Regulation, and Host Interactions

Classes of virulence determinants recur across bacterial pathogens, illustrating how genomic data informs mechanistic understanding. Genomic data identifies candidate virulence determinants, but mechanistic inference requires integration with functional data (transcriptomics, proteomics, and in vivo models) because expression and regulatory context determine clinical impact. Longitudinal and host-paired studies are particularly valuable for distinguishing colonization from invasive behavior and for validating genomic risk markers.

### 5.4. Clinical Correlates: Predicting Severity, Tropism, and Transmission Potential

Translating genomic virulence signatures into clinically useful predictions is an active area of translational research. Here, we review characteristics associated with specific clinical syndromes, such as an increased risk of hepatic abscess or metastatic spread, as well as traits influenced by genetic markers or combinations, capsule types, siderophore repertoires, and regulatory genes that enhance mucoviscosity [47]. The widespread utility of this approach may allow categorizing isolates as higher- or lower-risk for infection, while enhancing empirical decision-making, infection control prioritization, and public health responses through genomic risk scores that aggregate the presence/absence of multiple virulence loci. Some genomic signatures show associations with severity in retrospective cohorts, but predictive performance varies by species and clinical context. Prospective validation with host metadata is required before genomic virulence scores inform individual patient care.

### 5.5. Translational Potential and Current Limitations

The rational design of antivirulence therapeutics, of monoclonal antibodies, and vaccines is facilitated by the genomic mapping of conserved virulence determinants. Thus, conserved surface antigens and key virulence enzymes are rational vaccine targets; in contrast, anti-virulence drugs that interfere with siderophore systems, capsule assembly, or secretion systems might reduce pathogenicity while avoiding the selective pressure characteristic of bactericidal compounds [48]. More specifically, genomics enables reverse vaccinology by identifying and designing conserved, surface-exposed proteins shared across clinically relevant lineages. Several limitations temper these opportunities. High variability in the accessory genome limits vaccine coverage, and antigenic diversity or phase variation can lead to immune escape. If the targeted virulence factor is absent or easily lost in many clinical strains (i.e., it is accessory rather than essential), the effectiveness of antivirulence interventions will be reduced, and such strategies may have limited utility [47,49]. Thus, successful translational programs require that target prevalence be defined through genomic surveillance, mechanistic relevance be established using functional validation, and population-level impact be predicted with epidemiological modeling [50,51].

## 6. Precision Medicine and Clinical Integration

Genome sequencing not only enhances our understanding of pathogen biology but also has the potential to directly influence patient care. The coupling of sequencing with precision medicine refocuses the approach to infectious disease management from a broad population-based approach to an intervention strategy reserved for carefully defined strata, defined by the pathogen genotype, patient risk profile, and epidemiological context. This includes a discussion of the utility of sequencing data in personalized therapy, biomarker-driven management, and public health decision-making, as well as the hurdles that need to be overcome to implement them as part of standard clinical practice [52,53].

### 6.1. Individualized Therapy and Tailored Antimicrobial Selection

Guiding antimicrobial treatment is the most direct clinical application of sequencing-based precision medicine. WGS or mNGS can simultaneously identify species and detect resistance genes or mutations, thereby predicting antimicrobial susceptibility. When delivered within clinically actionable windows through validated pipelines, genomic information can guide timely de-escalation of empiric broad-spectrum therapy and the selection of narrow-spectrum agents. In aggregate, earlier genomic-informed adjustments may reduce inappropriate antimicrobial exposure and support stewardship; however, these benefits require local validation linked to clinical outcomes [54,55,56].

### 6.2. Biomarker-Guided Management and Public Health Decision

**Figure 4:**
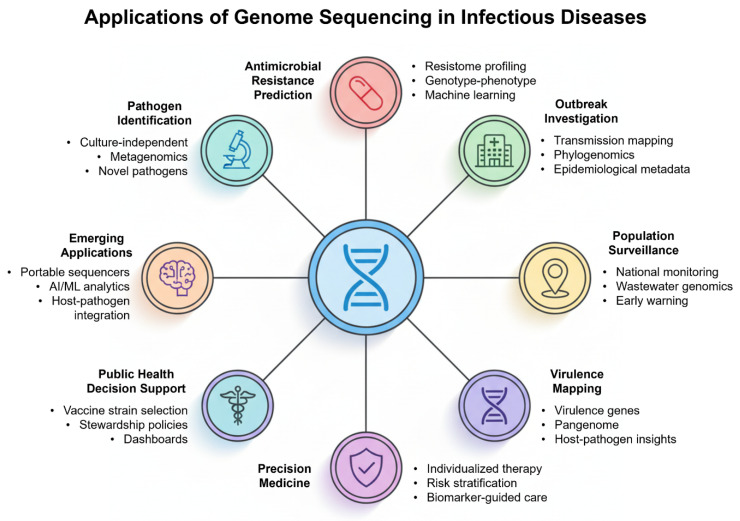
Applications of genome sequencing in infectious diseases. A hub-and-spoke schematic highlights diverse roles of sequencing, including pathogen identification, AMR prediction, outbreak investigation, population surveillance, virulence mapping, precision medicine, and public health decision support. Together, these applications demonstrate how sequencing bridges laboratory genomics with patient care and population health. “Novel pathogens” refers to newly emerging or previously uncharacterized strains associated with outbreaks or unexplained clinical syndromes; this includes both new species and novel genomic variants of known pathogens.

Predictive biomarkers, combined with sequences that are above single-pathogen calls, can also be generated during sequencing. Information on pathogen lineages may inform the likelihood of treatment failure, relapse risk, or transmission capacity. Integration of pathogen genomics with host-response biomarkers (transcriptomics/proteomics) can improve risk stratification and identify patients who may benefit from adjunctive therapies. Early clinical applications should prioritize robust, prospectively validated biomarkers with clear clinical actionability [57,58,59]. At the population level, precision medicine in infectious diseases intersects with public health. The ability of sequencing-informed risk stratification to inform isolation precautions, cohorting decisions, or prioritization of prophylaxis in exposed contacts can directly support infection-control and public health decision-making [60]. Genomic-informed risk stratification thereby supports targeted public health actions while minimizing unnecessary restrictions, but this requires integrated clinical–public health data flows and governance. Effective translation therefore depends on multidisciplinary networks that link genomic laboratories, informatics teams, clinicians, and public health authorities (Figure 4).

## 7. Challenges, Limitations, and Ethical Considerations

A combination of technical, operational, interpretive, regulatory, and ethical barriers limits the translation of genomic data from the research bench and sentinel surveillance into clinical standard of care. These limitations collectively determine whether sequencing yields actionable clinical insights or introduces uncertainty.

### 7.1. Technical and Interpretive Limitations

Low pathogen load and excess host nucleic acid in blood, CSF, and respiratory samples often reduce the number of informative reads, limiting reliable pathogen detection and genotyping. While library preparation and enrichment strategies address this issue, they only partially mitigate it; enrichment can introduce biases, and targeted assays may lead to false negatives as primer or probe sites evolve [61]. Nucleic acid detection alone cannot differentiate between live and dead organisms or determine whether a detected organism is the causative agent, a colonizer, or an environmental contaminant. Therefore, it requires careful integration with clinical, radiological, and laboratory data. Interpretive challenges compound technical limitations. Many organism-drug pairs lack genotype-to-phenotype relationships, and existing reference databases often lag emerging mechanisms and differ in terminology and scope. Insights into AMR mechanisms and epidemiology. Plasmid mobility and horizontal gene transfer decouple resistance determinants from chromosomal phylogeny, complicating inference about transmission and about which organism within a polymicrobial specimen carries a given resistance gene [26]. Within-host diversity and heteroresistance compound the confusion: minority resistant subpopulations relevant to the clinical phenotype of an infection may remain undetectable by standard sequencing techniques due to their low frequency.

### 7.2. Operational, Regulatory, and Economic Barriers

Sequencing in acute infections must deliver results within 24–48 h, but rapid turnaround requires dedicated laboratory capacity and automation. Although rapid workflows are possible, they often require dedicated laboratory capacity, automated library preparation, and on-site computational resources. Outsourcing to reference laboratories reduces the capital burden but usually introduces unacceptable delays for rapid clinical decision-making. Regulatory frameworks and laboratory accreditation represent additional hurdles. Performance attributes of sequencing tools, including validation metrics for sequencing assays, analytical sensitivity, specificity, reproducibility, and clinical concordance with phenotypic standards, must be established and consistently maintained under a quality management system. Sequencing is currently treated by many regulatory authorities as a diagnostic procedure, with the stringency of controls applied to other established clinical assays. However, guidance continues to change and varies from jurisdiction to jurisdiction. The policies on reimbursement are lagging behind the capability; without predictable funding models, institutions may be unable to internalize the costs of equipment, consumables, informatics infrastructure, and specialist personnel required to sustain service delivery [62,63]. Sustained adoption will depend on coordinated investment in regional sequencing hubs, clear regulatory guidance, and predictable reimbursement models.

### 7.3. Ethical, Legal, and Social Implications

Pathogen genomic data are often accompanied by sensitive metadata, including collection dates, locations, and clinical context, which can inadvertently lead to the re-identification of individuals or institutions if appropriate governance frameworks are not in place. To safeguard privacy while maintaining data utility, institutions should implement a layered governance structure that includes minimal metadata sharing for public repositories, controlled access to sensitive linkages, such as patient identifiers, through data access committees, and the adoption of federated or privacy-preserving analytical approaches that enable model training on local data without bulk data transfers. Consent models must be pragmatic and context-specific: while routine diagnostic sequencing should be governed institutionally to allow for essential public health surveillance, secondary research applications should follow opt-in or broad consent principles supported by clear benefit-sharing mechanisms [64,65]. Equitable implementation in low- and middle-income countries requires investment in local capacity, fair data-sharing agreements, and recognition of data ownership [66,67,68]. Practical ways to enable privacy-respecting collaborative surveillance include federated data models, harmonized consent models, and privacy-preserving analytics. Embedding sequencing within multidisciplinary teams linking clinicians, microbiologists, bioinformaticians, and ethicists ensures responsible interpretation and patient-centered use.

## 8. Future Perspectives

Genome sequencing of infectious diseases has reached a level of maturity in which many of the obstacles to its larger-scale adoption are less technical than they are organizational, interpretive, and social. Advances in portable platforms, automation, and AI-assisted analytics will enable faster, bedside sequencing with real-time interpretation [1,69]. Machine-learning tools will complement rule-based frameworks if training data and validation metrics are transparent. Interoperable standards and privacy-preserving data systems are essential for secure, cross-jurisdictional sharing [1,70]. Global equity requires affordable platforms, regional hubs, and public–private partnerships to sustain reagent supply and workforce training. Also, data systems architecture and governance will be critical. Responsible genomic practice demands external quality assurance, standardized reference materials, and transparent governance so that sequencing becomes a routine, safe, and equitable component of infectious-disease care.

## 9. Conclusions

Genome sequencing is poised to transition from a specialized research tool to a cornerstone of clinical and public health practice, but achieving this shift demands coordinated, multi-level action. Sequencing provides information that was inconceivable even ten years ago, from identifying previously unrecognized pathogens to defining transmission networks with unprecedented resolution. Together with recent advances in bioinformatics and systems biology, it provides a potent framework for a better understanding and management of AMR, virulence, and host–pathogen interactions. Standardization and validation of sequencing workflows are essential, requiring the adoption of accredited, version-controlled pipelines, rigorous external quality assessment, and transparent documentation of analytical provenance. A staged approach to implementation should be pursued, starting with applications in pathogen surveillance and infection control, and then extending to therapeutic decision support once local validation and phenotypic confirmation are established. Interoperability must also be prioritized through the development of standardized APIs and report formats that allow seamless integration of concise genomic insights into electronic health records and antimicrobial stewardship systems. Sustainable adoption will further depend on investment in workforce development, regional sequencing hubs, and reliable reagent supply chains to ensure equitable access, particularly across low- and middle-income regions. Collectively, transparent governance, validated machine-learning frameworks, and federated data-sharing models will ensure that genome sequencing becomes a safe, equitable, and clinically meaningful component of infectious-disease medicine.

## Figures and Tables

**Table 1:** Comparative summary of sequencing platforms for infectious disease applications *.

Technology	Platform Examples	Typical Read Length	Accuracy (Per Base)	Throughput (Per Run)	Strengths	Limitations	Representative Applications
Short-read sequencing	Illumina MiSeq, NextSeq, NovaSeq	50–300 bp (paired-end)	>99.9%	Up to hundreds of Gb, depending on system	High accuracy, cost-effective, standardized pipelines	Fragmented assemblies in repetitive regions; poor plasmid/structural resolution	Routine bacterial WGS, viral surveillance, variant calling
Long-read sequencing (Nanopore)	ONT MinION, GridION, PromethION	1 kb to several Mb	~90–99% (raw)	Tens to hundreds of Gb	Real-time sequencing, portable, long contigs	Lower per-base accuracy; error-prone homopolymers	Rapid pathogen ID, outbreak response, metagenomics
Long-read sequencing (PacBio HiFi)	Sequel IIe, Revio	15–20 kb (up to 50 kb)	>99.9%	~60–120 Gb	Highly accurate long reads, excellent assembly quality	Higher cost per run; longer prep workflows	Complete assemblies, plasmid and resistance island mapping
Targeted sequencing	Ion Torrent, AmpliSeq panels	200–600 bp	~98–99%	Few Gb	Fast turnaround, focused panels, high sensitivity for known loci	Requires prior knowledge; limited to predefined targets	AMR gene panels, viral genotyping, clinical diagnostics

* Short-read Illumina systems provide high accuracy at modest read lengths, while Nanopore and PacBio deliver long reads that enable structural resolution and plasmid assembly. Targeted approaches complement these methods by enabling rapid, sensitive detection of predefined loci.

**Table 2:** Integration of genome sequencing into clinical diagnostic workflows *.

Clinical Stage	Conventional Approach	Sequencing-Enhanced Approach	Advantages of Sequencing	Challenges
Pathogen identification	Culture, microscopy, antigen tests	Shotgun metagenomic sequencing	Culture-independent, hypothesis-free, broad detection	Requires sufficient pathogen load; host DNA background
Resistance profiling	Phenotypic AST (growth-based)	Whole-genome sequencing (resistome analysis)	Faster turnaround, multiple resistance determinants at once	Novel/unknown mechanisms may be missed; interpretation complexity
Outbreak investigation	PFGE, MLST, serotyping	Whole-genome sequencing (phylogenomics)	Ultra-fine resolution enables transmission mapping	Requires bioinformatics infrastructure; careful interpretation
Treatment decision-making	Empirical therapy based on clinical suspicion	Genomic-informed therapy (ID + AMR prediction)	Enables precision prescribing, stewardship optimization	Integration into clinical workflows, need for rapid turnaround

* Sequencing augments or replaces conventional methods at multiple stages of care, enabling culture-independent detection, rapid resistance prediction, high-resolution outbreak analysis, and more precise therapeutic guidance, while challenges remain in turnaround time, interpretation, and implementation.

**Table 3:** Representative tools and databases for genomic AMR prediction *.

Tool/Database	Methodology	Detects	Key Features	Limitations
ResFinder	BLAST search against curated gene sets	Acquired resistance genes	Web-based, easy-to-use, regularly updated	Limited to novel/rare mechanisms
CARD (RGI)	BLAST + HMM models	Resistance genes, allelic variants, mutations	Broad ontology; includes efflux, enzymatic, and regulatory mechanisms	Requires expertise to interpret outputs
AMRFinderPlus	BLAST + HMMs	Genes and key resistance mutations	Integrated with NCBI pathogen pipelines; curated reference sets	Primarily bacterial pathogens; limited viral coverage
ARIBA	Local de novo assembly + alignment	Genes and alleles (customizable)	Flexible use with custom databases, suitable for local validation	Requires high-quality assemblies; more complex setup

* Widely used platforms such as ResFinder, CARD, AMRFinderPlus, and ARIBA employ different computational strategies and curated datasets to identify acquired genes and resistance-associated mutations. Each provides complementary strengths for surveillance and clinical application.

## Data Availability

No new data were created or analyzed in this study.

## Abbreviations

AI—Artificial Intelligence; AMR—Antimicrobial Resistance; ANI—Average Nucleotide Identity; CDC—Centers for Disease Control and Prevention; cgMLST—Core Genome Multi-Locus Sequence Typing; CNV—Copy Number Variation; CSF—Cerebrospinal Fluid; ddPCR—Droplet Digital PCR; DNA—Deoxyribonucleic Acid; DNA-seq—DNA Sequencing; EHR—Electronic Health Records; HGT—Horizontal Gene Transfer; HIFI—High-Fidelity (long reads); HIV—Human Immunodeficiency Virus; ICU—Intensive Care Unit; INDEL—Insertion/Deletion; LIMS—Laboratory Information Management System; LOD—Limit of Detection; MDR—Multidrug-Resistant; ML—Machine Learning; MLST—Multi-Locus Sequence Typing; mNGS—Metagenomic Next-Generation Sequencing; NC—Negative Control; NGS—Next-Generation Sequencing; ONT—Oxford Nanopore Technologies; ORF—Open Reading Frame; PacBio—Pacific Biosciences; PDR—Pan-Drug-Resistant; PCR—Polymerase Chain Reaction; qPCR—Quantitative Polymerase Chain Reaction; QA—Quality Assurance; QC—Quality Control; RNA—Ribonucleic Acid; RNA-seq—RNA Sequencing; rRNA—Ribosomal RNA; RT-PCR—Reverse Transcription Polymerase Chain Reaction; SARS-CoV-2—Severe Acute Respiratory Syndrome Coronavirus 2; SNP—Single-Nucleotide Polymorphism; SNV—Single-Nucleotide Variant; ST—Sequence Type; WGS—Whole-Genome Sequencing; WHO—World Health Organization; XDR—Extensively Drug-Resistant.
